# *Bacillus coagulans* restores pathogen-induced intestinal dysfunction via acetate–FFAR2–NF-κB–MLCK–MLC axis in *Apostichopus japonicus*

**DOI:** 10.1128/msystems.00602-24

**Published:** 2024-06-28

**Authors:** Mingshan Song, Shanshan Zhang, Zhen Zhang, Liyuan Guo, Weikang Liang, Chenghua Li, Zhonghua Wang

**Affiliations:** 1 State Key Laboratory for Managing Biotic and Chemical Threats to the Quality and Safety of Agro-products, Ningbo University, Ningbo, China; 2 Laboratory for Marine Fisheries Science and Food Production Processes, Qingdao National Laboratory for Marine Science and Technology, Qingdao, China; 3 Shandong Beiyou Biotechnology Co.，Ltd., Weifang, China; University of Technology Sydney, Glebe, Australia

**Keywords:** intestinal microbiota, intestinal microbiota transplantation, *Bacillus coagulans*, acetate, intestinal barrier

## Abstract

Skin ulceration syndrome (SUS) is currently the main disease threatening *Apostichopus japonicus* aquaculture due to its higher mortality rate and infectivity, which is caused by *Vibrio splendidus*. Our previous studies have demonstrated that SUS is accompanied by intestinal microbiota (IM) dysbiosis, alteration of short-chain fatty acids (SCFAs) content and the damage to the intestinal barrier. However, the mediating effect of IM on intestine dysfunction is largely unknown. Herein, we conducted comprehensive intestinal microbiota transplantation (IMT) to explore the link between IM and SUS development. Furthermore, we isolated and identified a *Bacillus coagulans* strain with an ability to produce acetic acid from both healthy individual and SUS individual with IM from healthy donors. We found that dysbiotic IM and intestinal barrier function in SUS recipients *A. japonicus* could be restored by IM from healthy donors. The *B. coagulans* strain could restore IM community and intestinal barrier function. Consistently, acetate supply also restores intestinal homeostasis of SUS-diseased and *V. splendidus*-infected *A. japonicus*. Mechanically, acetate was found to specifically bind to its receptor-free fatty acid receptor 2 (FFAR2) to mediate IM structure community and intestinal barrier function. Knockdown of FFAR2 by transfection of specific FFAR2 siRNA could hamper acetate-mediated intestinal homeostasis *in vivo*. Furthermore, we confirmed that acetate/FFAR2 could inhibit *V. splendidus*-activated NF-κB–MLCK–MLC signaling pathway to restore intestinal epithelium integrity and upregulated the expression of ZO-1 and Occludin. Our findings provide the first evidence that *B. coagulans* restores pathogen-induced intestinal barrier dysfunction via acetate/FFAR2–NF-κB–MLCK–MLC axis, which provides new insights into the control and prevention of SUS outbreak from an ecological perspective.

**IMPORTANCE:**

Skin ulceration syndrome (SUS) as a main disease in *Apostichopus japonicus* aquaculture has severely restricted the developmental *A. japonicus* aquaculture industry. Intestinal microbiota (IM) has been studied extensively due to its immunomodulatory properties. Short-chain fatty acids (SCFAs) as an essential signal molecule for microbial regulation of host health also have attracted wide attention. Therefore, it is beneficial to explore the link between IM and SUS for prevention and control of SUS. In the study, the contribution of IM to SUS development has been examined. Additionally, our research further validated the restoration of SCFAs on intestinal barrier dysfunction caused by SUS via isolating SCFAs-producing bacteria. Notably, this restoration might be achieved by inhibition of NF-κB–MLCK–MLC signal pathway, which could be activated by *V. splendidus*. These findings may have important implications for exploration of the role of IM in SUS occurrence and provide insight into the SUS treatment.

## INTRODUCTION

Intestinal microbiota (IM) exerts essential roles in regulating host health, metabolism, diseases, and other physiological processes (1, 2). A large number of studies on human disease have demonstrated that the natural homeostasis of intestinal microbial communities dramatically changes during various disease pathologies including obesity, diabetes, inflammatory bowel disease (IBD), and inflammatory bowel syndrome (IBS) (3–5). To some extent, many complex diseases have been linked to microbial alterations, and aquatic animal diseases are no exception (6). During the last decade, the importance of IM in aquatic animals has been highlighted, and it is currently considered an adjunct organ due to the abundance of microbial cells and their effect on the physiological processes of a large number of aquatic animals. Several studies have demonstrated the close relationship between intestinal microbial community and disease in fish (7). The performance of fish and the production of aquaculture can be affected by the dysbiosis of IM (8, 9). Recent reviews suggested that diseases will still be one of the main risks for aquaculture in the near and mid-term future (10). However, the studies investigating links between the microbiota and host diseases have mainly focused on fish, but the echinoderms. Increasing evidence showed that the IM of white feces syndrome (WFS)-affected shrimp is less diverse and significantly different from those of healthy shrimp, and WFS in shrimp is correlated with dysbiosis in IM (11–13).

Modulation of the IM has been studied extensively in diseases of various mammals (14, 15). Therefore, utilizing the IM regulation in host could provide an attractive preventive and therapeutic opportunity for several diseases. Currently, intestinal microbiota transplantation (IMT) has become an efficient treatment applied in multiple fields such as enteritis, metabolic diseases, and autoimmune diseases. It is mainly realized through reestablishment of a healthy gut microbial community and inhibition of pathogens growth in humans, mice, pigs, and other mammals (16–20). IMT for diseases rapidly restores a gut microbiota, back toward pre-morbid composition and diversity, which resembles that of healthy donors (21). Interest in the potential application of IMT for a range of aquatic animal diseases is also growing. The colonization of intestinal flora community from young individuals successfully spans the life of middle-aged individuals in African turquoise killifish (22). Huang et al (6) also demonstrated that WFS in shrimp can be cured by IMT. The sea cucumber *Apostichopus japonicus* is an important marine economic species in Asian countries due to its profound nutritional and medicinal value. Skin ulceration syndrome (SUS) induced by *V. splendidus* is the main disease threatening *A. japonicus* production, which has been widely discussed. The symptoms of SUS are ulcers on the mouth and body wall of diseased *A. japonicus*, with blue and white spots forming at the ulceration site. The diseased *A. japonicus* eventually delinquents and dies. Our previous studies have demonstrated that SUS occurrence is accompanied by dysbiosis of IM in *A. japonicus* (23, 24). Therefore, restoring IM health of *A. japonicus* may become a new strategy for preventing and treating SUS.

A key area of interest has been the potential mechanism of IMT through the restoration of microbial metabolites (21). IM contributes to the physiological processes of the host through the production of a myriad of metabolites. One class of metabolites well-studied in this field are the short-chain fatty acids (SCFAs), which come from indigestible carbohydrates (25). Our earlier work has reported that levels of a range of SCFAs within gut content (including acetate, propionate, butyrate, valerate, and caproate) are very low in SUS-diseased *A. japonicus* (24). In these experiments, certain SCFAs (acetate) can improve the intestinal barrier function of SUS-diseased *A. japonicus*, which is consistent with the results in other reports (26). Some studies have also demonstrated that the successful IMT for disease in humans is associated with rapid, sustained restoration of SCFAs to levels comparable to healthy donors (27, 28). Restoration of SCFAs by IMT may allow the host to recover from the diseases by mechanisms, which inhibit the growth of pathogen, including a possible role in the resolution of intestinal immune response and gut barrier function (21). Thus, harnessing the SCFAs restoration properties of the IMT to prevent *A. japonicus* SUS in *A. japonicus* needs further investigation, given that the SCFAs administration of SCFAs is liable to a dose-dependent.

The barrier function of epithelial cells is the first line of defense in the gut (29). Although, it is well established that SCFAs are the most important components for maintaining the integrity of the intestinal barrier, the mechanism by which SCFAs regulate intestinal barrier function is still unclear (30). The specific effect of SCFAs is largely mediated by selective activation of free fatty acid 2 (FFAR2) (31, 32). Multiple signaling pathways are associated with the barrier dysfunction. Myosin light-chain kinase (MLCK) signaling pathway regulates tight junction (TJ) structure via expression and assembly of TJ proteins. The MLCK signaling pathway can be activated by phosphorylation of NF-κB p50/p65 dimer. Then, the activated MLCK can induce the myosin light-chain (MLC) phosphorylation that directly downregulates the expression of structural-functional proteins in intestinal barrier and results in increasing the barrier permeabilization (33, 34). Pan et al. (35) suggested that berberine can repair intestinal mucosal damage in patients with inflammatory diarrhea via suppression of MLCK–MLC phosphorylation signaling pathway. Therefore, we hypothesize that SCFAs upregulate the expression level of ZO-1 and Occludin by suppressing NF-κB–MLCK–MLC signaling pathway to protect intestinal epithelial integrity.

In this study, we investigated the link between IM dysbiosis and SUS development which was first validated by reciprocal IMT in *A. japonicus*. The single bacterium producing acetic acid was transplanted in *A. japonicus* as probiotics to verify the effect of acetate on regulating the intestine barrier function. In addition, the mechanisms by which acetate as intermediates regulates intestinal barrier function were discussed. These valuable findings greatly enhance our understanding of the interaction of the host and IM and provide us with new strategies for preventing and treating SUS from the perspective of intestinal microecology. In addition, we investigated whether FFAR2–NF-κB–MLCK–MLC could be involved in these negative effects of *V. splendidus* and positive effects of acetate by transfection of siRNA FFAR2. Our present study and findings may provide new insights into the role of microbiota metabolites in the intervention of SUS.

## RESULTS

### Intestine microbe contributes to SUS development by IMT

**Fig 1:**
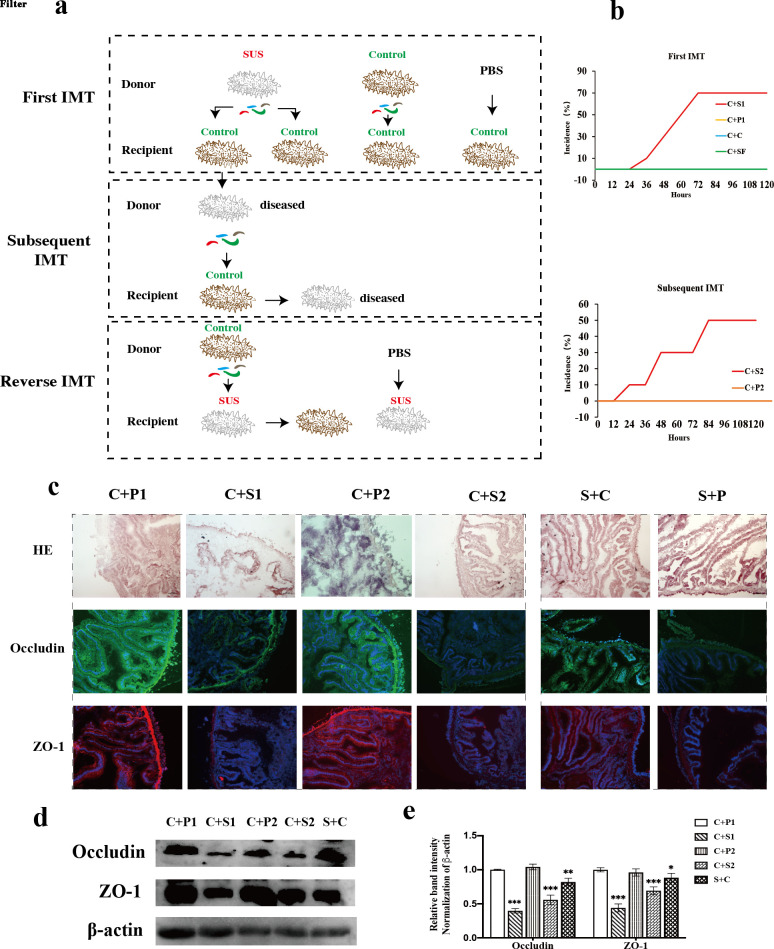
IMT leads to similar symptoms of SUS. (a) Schematic representation of IMT procedures. (b) Incidence of recipient *A. japonicus* suffered from SUS after the first IMT and the subsequent IMT. (c) Intestinal histological pathology and immunofluorescence of Occludin and ZO-1. (d, e) Western blotting of tight junction proteins in IMT *A. japonicus*. Significant differences are indicated by asterisks (*, *P* < 0.05; **, *P* < 0.01; ***, *P* < 0.001).

IM from SUS donors (*n* = 6) were transplanted to healthy *A. japonicus* (C + S1, *n* = 10) by IMT to demonstrate whether the aberration of IM is a causal factor in SUS occurrence *in vivo*. Seven recipients *A. japonicus* (70%) developed SUS signs in the first IMT experiment (Fig. 1a and b). At 36-h post-transplantation, one *A. japonicus* developed SUS signs. At 48 h, the number of SUS-diseased *A. japonicus* was 2. The number of SUS-diseased *A. japonicus* both 2 at 60 and 72 h after IMT (Fig. 1b; Table S1). However, recipients who received phosphate-buffered saline (PBS) (C + P1, *n* = 10) or IM of healthy donors (C + C, *n* = 10) did not cause SUS. Furthermore, healthy *A. japonicus* receiving IM filtrates from SUS donors (C + SF, *n* = 10) did not suffer from SUS, which indicates that the virus did not contribute to SUS. To further demonstrate whether the dysbiotic IM exists in the new SUS, seven SUS-diseased *A. japonicus* in the C + S1 group were selected as the donors for the subsequent IMT. Control *A. japonicus* samples were divided into two groups (*n* = 10) to receive IM (C + S2) and PBS (C + P2). Consequently, five recipients (50%) exhibited SUS symptoms after the second IMT (Fig. 1a and b; Table S1), as supported by progressive severity after IMT (Fig. 1). However, SUS did not occur in the C + P2 group. Although the two groups of IM community SUS-diseased *A. japonicus* were separately transferred by IMT with control donors (S + C) and PBS (S + P), of which no SUS-diseased *A. japonicus* in the S + C group recovered to health (Fig. 1b). Collectively, these findings demonstrate that dysbiosis IM causally contributes to SUS. The result of intestinal pathology and TJ protein immunofluorescence further confirmed that the IM from different donors had different effects on the intestinal barrier in *A. japonicus*. As shown in Fig. 1c, IM from SUS donors damaged the structure of epithelial tissues in the recipients *A. japonicus*, which led to striated bord detachment and reduction in mucosal integrity. Meanwhile, the expression of Occludin and ZO-1 significantly downregulated in the control recipients *A. japonicus* who received IM from SUS donors (C + S1, C + S2) compared with (C + P1, C + P2) as well. However, recipients *A. japonicus* who received PBS (C + P1, C + P2) possessed complete intestinal epithelial tissue (Fig. 1d and e). Intriguingly, the epithelial integrity and the expression level of Occludin and ZO-1 were recovered by normal IM in SUS recipients *A. japonicus*, although the SUS recipients *A. japonicus* did not restore health.

### IMT could rearrange the intestine microbe in recipient *A. japonicus*

**Fig 2:**
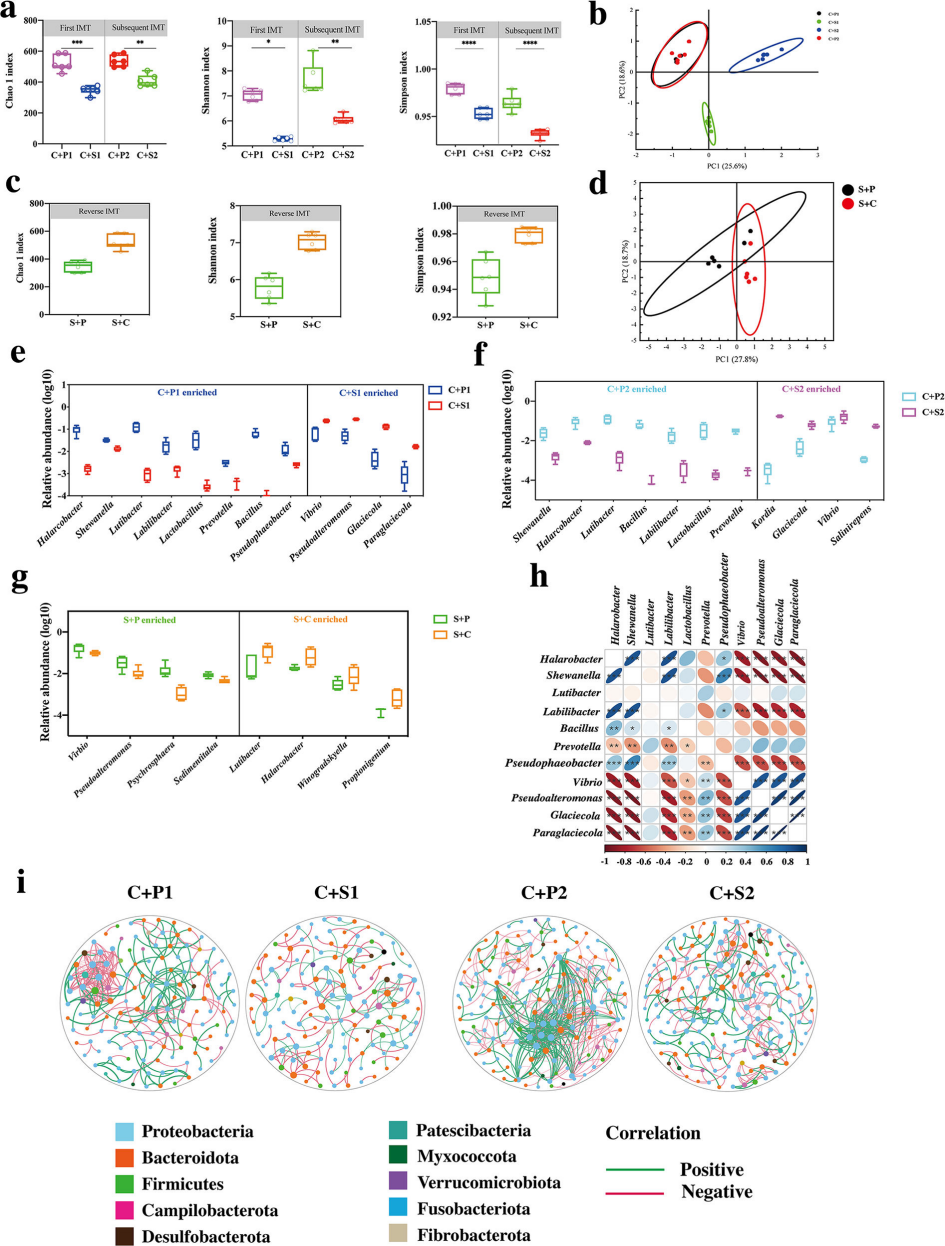
Recharacterize the IM dysbiosis in the newly diseased *A. japonicus*. (a) α-diversity (*P* < 0.05, Student’s *t* test) in the first IMT and subsequent IMT. (b) PCoA of first IMT and subsequent IMT. (c) α-diversity (*P* < 0.05, Student’s *t* test) in reverse IMT. (d) The PCoA of reverse IMT. (e–g) Boxplot comparing the abundance of altered genera after receiving the IMT and reverse IMT. (h) Relationship heatmap among the distinguished genera. *P* < 0.05 (Spearman’s correlation). (i) The molecular ecology networks (MENs) of different groups. Significant differences are indicated by asterisks (*, *P* < 0.05; **, *P* < 0.01; ***, *P* < 0.001).

**Fig 3:**
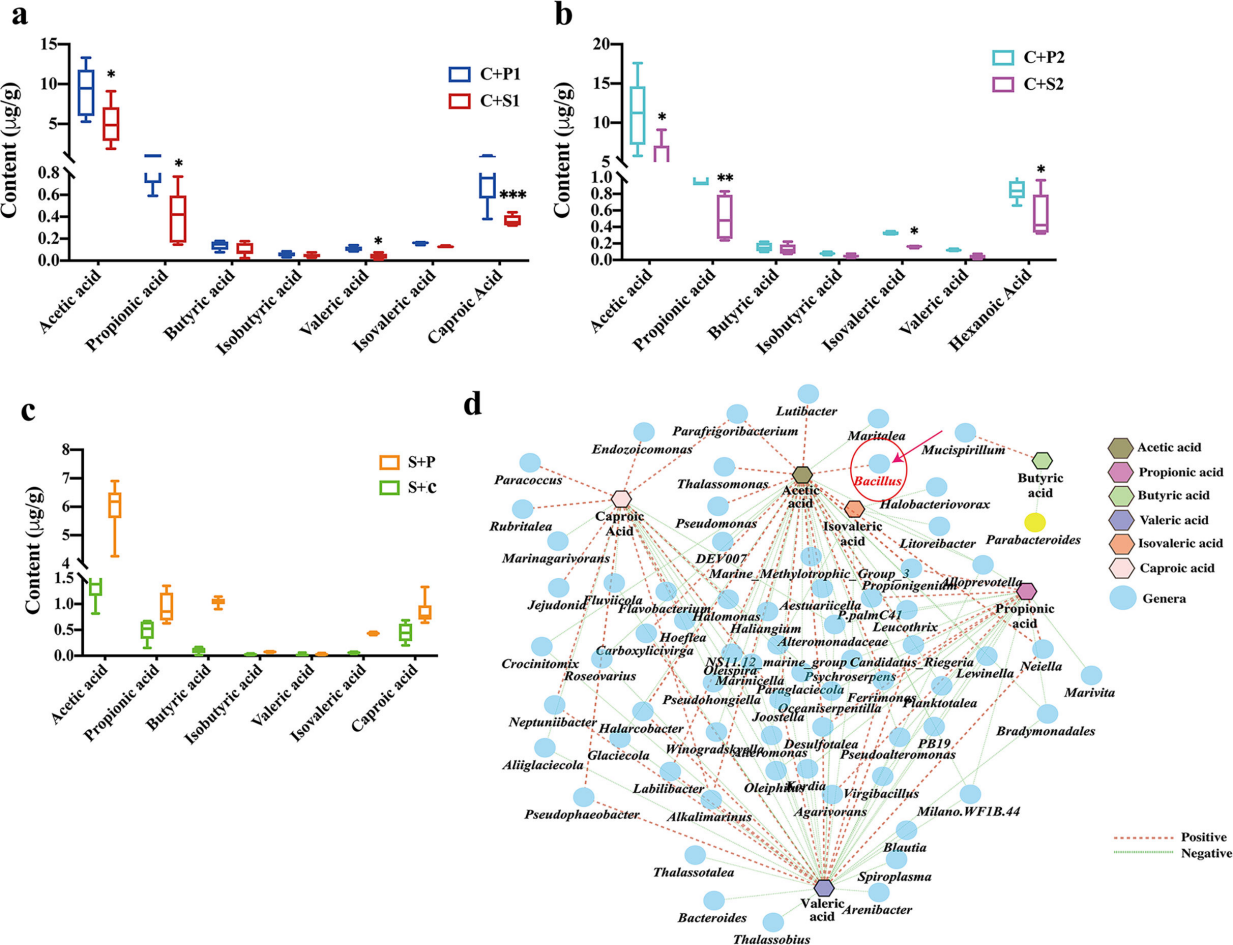
The impact of IMT on SCFAs contents in *A. japonicus*. (a) The seven SCFAs content decreased in first IMT. (b) The seven SCFAs content decreased in subsequent IMT. (c) The SCFAs content increased after receiving the reverse IMT. (d) The relationship of distinguished SCFAs and genera (Spearman’s correlation analysis, |*r*| > 0.7). Significant differences are indicated by asterisks (*, *P* < 0.05; **, *P* < 0.01; ***, *P* < 0.001).

We performed 16S rRNA gene high-throughput sequencing to characterize the IM community after IMT (PRJNA1047199) for further confirming IMT-mediated SUS development by rearranging IM community. The α-diversity in the C + S1 and C + S2 groups was significantly lower than that in the C + P1 and C + P2 ones (Fig. 2a). PCoA biplot revealed that samples from the C + S1 and C + S2 groups closely clustered and were distinct from those of control groups (C + P1 and C + P2, Fig. 2b). The results of reverse IMT showed that the IM of SUS recipient *A. japonicus* transplanted by control IM with higher α-diversity (Fig. 2c) to SUS-diseased *A. japonicus* and were distinct from these of SUS individuals (Fig. 2d). At the bacterial genus level, *Vibrio*, *Pseudoalteromonas*, *Glaciecola*, and *Paraglaciecola* were confirmed to be abundant in the C + S1 and C + S2 groups, while *Shewanella*, *Bacillus*, *Prevotella*, *Lactobacillus*, and *Lutibacter* were more deficient in the C + P1 and C + P2 groups (Fig. 2e and f). The reverse IMT increases the abundance of *Lutibacter* and *Propionigenium* and decrease the abundance of *Vibrio*, *Pseudoalteromona*, *Psychrosphaera*, and *Sedimentitalea* abundant (Fig. 2g). Furthermore, the SUS enriched *Vibrio*, *Pseudoalteromonas*, *Glaciecola*, and *Paraglaciecola* were negatively correlated with the control enriched genera (*Shewanella*, *Bacillus*, *Prevotella*, and *Halarobacter*) (Spearman’s correlation: *P* < 0.05, Fig. 3h). A molecular ecological network was constructed to understand species interactions of the IM after IMT (Fig. 2i). The SUS-related IM (SUS, C + S1, and C + S2) showed lower links and more negative relationship than the control-related IM (control, C + P1, and C + P2 groups), which indicates that species interactions of SUS were less co-operative and complex. In control-related networks, the majority of the nodes belonged to Proteobacteria species, while the major nodes in SUS shifted into Bacteroidota (Fig. 2i).

### Impact of IMT on intestinal SCFAs profiles

We examined the SCFAs profiles in recipient *A. japonicus* to further identify the regulation of IM on host health via SCFAs. As shown in Fig. 3, the SUS-related IM (SUS, C + S1, C + S2) showed lower acetic acid, propionic acid, isovaleric acid, and caproic acid content than the control-related IM (control, C + P1, and C + P2 groups). However, the reverse IMT significantly increases the content of acetic acid, propionic acid, butyric acid, isovaleric acid, and caproic acid in SUS recipients. Spearman’s correlation analysis depicted the relationship between 7 SCFAs and the 136 most different genera (Table S1). As shown in Fig. 3d; Table S1, there were significant associations existed between IM and SCFAs, including acetic acid associated with the greatest number of genera that are potential probiotics, such as *Bacillus* (*r* = 0.755, *P* < 0.01), and negatively correlated with some of potential pathogens, such as *Pseudoalteromonas* (*r* = −0.804, *P* < 0.01) and *Glaciecola* (*r* = −0.797, *P* < 0.01). In addition, the relationships of propionic acid and *Propionigenium* (*r* = 0.753, *P* < 0.01), as well as between caproic acid and *Pseudophaeobacter* (*r* = 0.914, *P* < 0.001), were all negatively correlated. Butyric acid was only associated with two species genera, namely, *Mucispirillum* and *Parabacteroides* (*r* = 0.758, *P* < 0.01; *r* = 0.776, *P* < 0.01).

### *Bacillus coagulans* isolated from healthy IM elevates the acetate level and restores intestinal dysfunction

**Fig 4:**
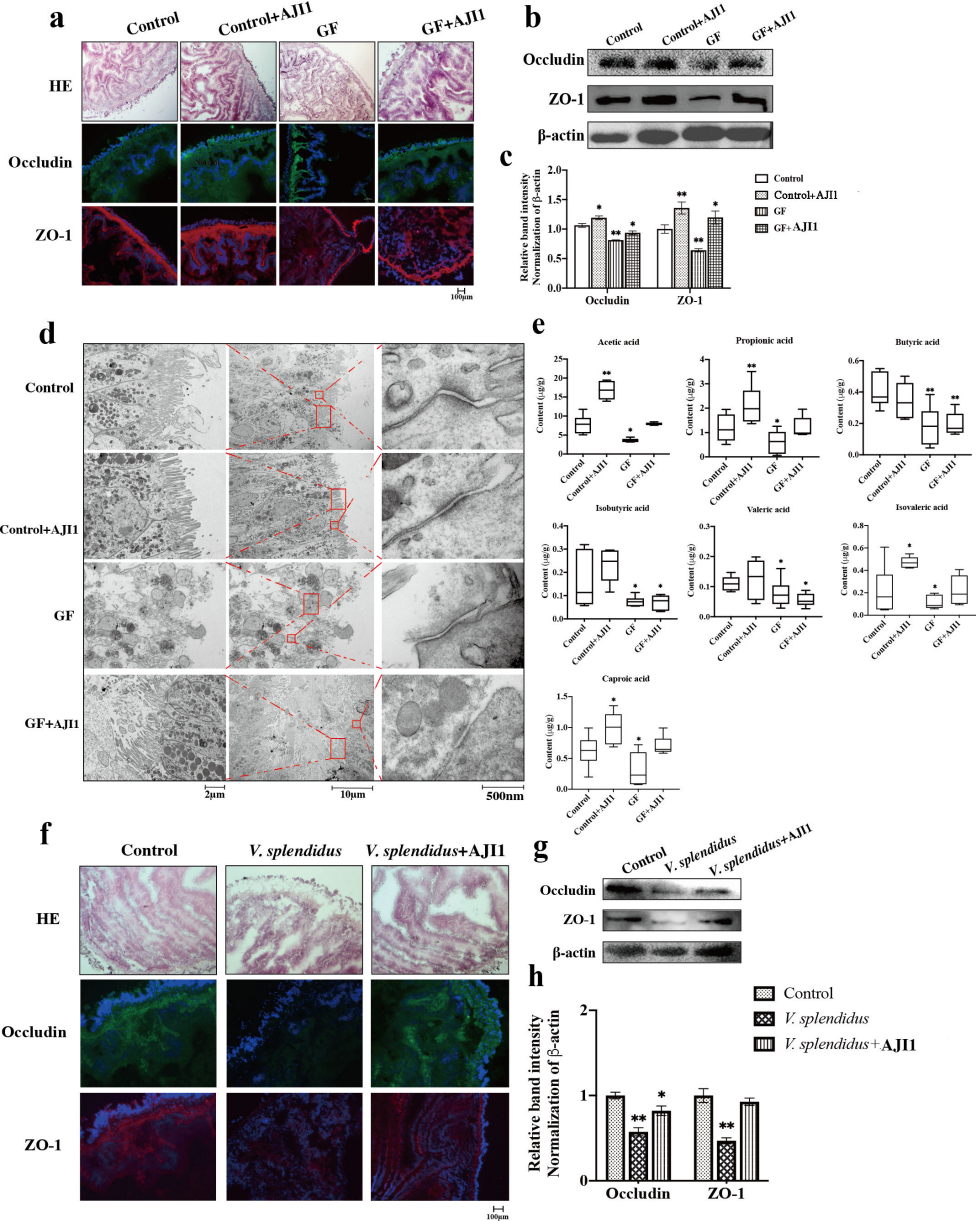
The *Bacillus coagulans* AJI1 exhibits protective effect on epithelial integrity and increases the content of acetic acid. (a) Intestinal histological pathology and immunofluorescence of Occludin and ZO-1 in control and GF *A. japonicus* treated with bacterial strain *B. coagulans* AJI1. (b, c) Western blotting of tight junction proteins. (d) Representative transmission electron microscopy (TEM) micrographs show the effect of *B. coagulans* AJI1 on intestine tight junction structure in control and GF *A. japonicus*. (e) The seven SCFAs contents in control and GF *A. japonicus* treated with bacterial strain *B. coagulans* AJI1. (f) Intestinal histological pathology and immunofluorescence of Occludin and ZO-1 show the mitigative effect of *B. coagulans* AJI1 on tight junction structure under *V. splendidus* stress. Significant differences are indicated by asterisks (*, *P* < 0.05; **, *P* < 0.01; ***, *P* < 0.001).

We examined the roles of individual bacterial strains producing acetic acid on epithelial integrity to further verify the protective effect of acetate on intestinal barrier (30, 36). After enrichment cultivation and purification cultivation, we isolated one *Bacillus coagulans* strain from the adult *A. japonicus* intestine and sequenced the full-length 16S rRNA gene to confirm its taxonomy classification, and it was referred to as *B. coagulans* AJI1 (Fig. S1). To define the intestinal barrier protective function, *B. coagulans* AJI1 was further transplanted into the intestines of normal *A. japonicus* (control) and germ-free GF *A. japonicus* intestine (Fig. S2). The result revealed that *B. coagulans* AJI1 not only contributed to the restoration of intestinal morphology, but also increased the expression level of TJ (Fig. 4a through c). Normal *A. japonicus* inoculated *B. coagulans* AJI1 possessed more complete intestinal tissue structure, more uniform and orderly arrangement of epithelial cells, and thicker and more continuous muscle layer than control *A. japonicus*. The intestinal villi in GF *A. japonicus* were damaged and shed, and the muscle layer became thinner and broken. After *B. coagulans* AJI1 inoculation, GF *A. japonicus* villus relatively became complete (Fig. 4a). Occludin and ZO-1 mainly distributed on the surface of the intestinal mucosa and in the crypts of the intestinal mucous. The immunofluorescence results showed that *B. coagulans* AJI1 inoculation induced relatively dense distribution of Occludin and ZO-1, and the expression level of Occludin and ZO-1 was significantly higher than those in control *A. japonicus* (Fig. 4a through c). The distribution of Occludin and ZO-1 relatively dispersed in GF *A. japonicus*, but their expression level significantly increased after AJI1 inoculation (Fig. 4a through c). The observation of ultrastructure by electron microscopy showed that villi on the surface of intestine in GF *A. japonicus* were not only few and scattered but also uneven. They also exhibited obvious disarrangement, inordinate array, and extensive absence. Meanwhile, tight junction structure separation and looseness of TJ structure were observed between epithelial cells (Fig. 4d). However, intestinal ultrastructure in the control, control + AJI1 and GF + AJI1 groups did not obviously change, and TJ structure was normal (Fig. 4d).

To further demonstrate the protective effect of acetate on intestinal homeostasis, we colonialized AJI1 in the intestines of control and GF *A. japonicus* intestine. We first measured the SCFAs content in *A. japonicus* intestine before and after AJI1 inoculation. The result indicated that AJI1 inoculation significantly increased the content of acetic acid in control *A. japonicus* (*P* < 0.01), while AJI1 did not significantly affect the content of butyric acid, isobutyric acid, and valeric acid. Intestinal bacterial clearance significantly affected the SCFAs production, seven of which reached the lowest level in GF *A. japonicus* among the four treatments (*P* < 0.05). However, AJI1 could restore the content of acetic acid, propionic acid, isovaleric acid, and caproic acid to the control level in GF *A. japonicus* (Fig. 4e).

Then, we examined whether *B. coagulans* AJI1 could alleviate damage to intestinal barrier function induced by *V. splendidus* infection. As shown in Fig. 4f, AJI1 colonization recovered restructuration of intestinal epithelium caused by *V. splendidus*. The immunochemical and Western blot analysis results suggested that AJI1 significantly increased the expression level of Occludin and ZO-1 compared with *V. splendidus* infection group to attenuated *V. splendidus*-induced intestinal barrier dysfunction (Fig. 4f through h).

### Acetate mediated a protective effect on intestinal barrier via depression FFAR2–NF-κB–MLCK–MLC signal pathway

**Fig 5:**
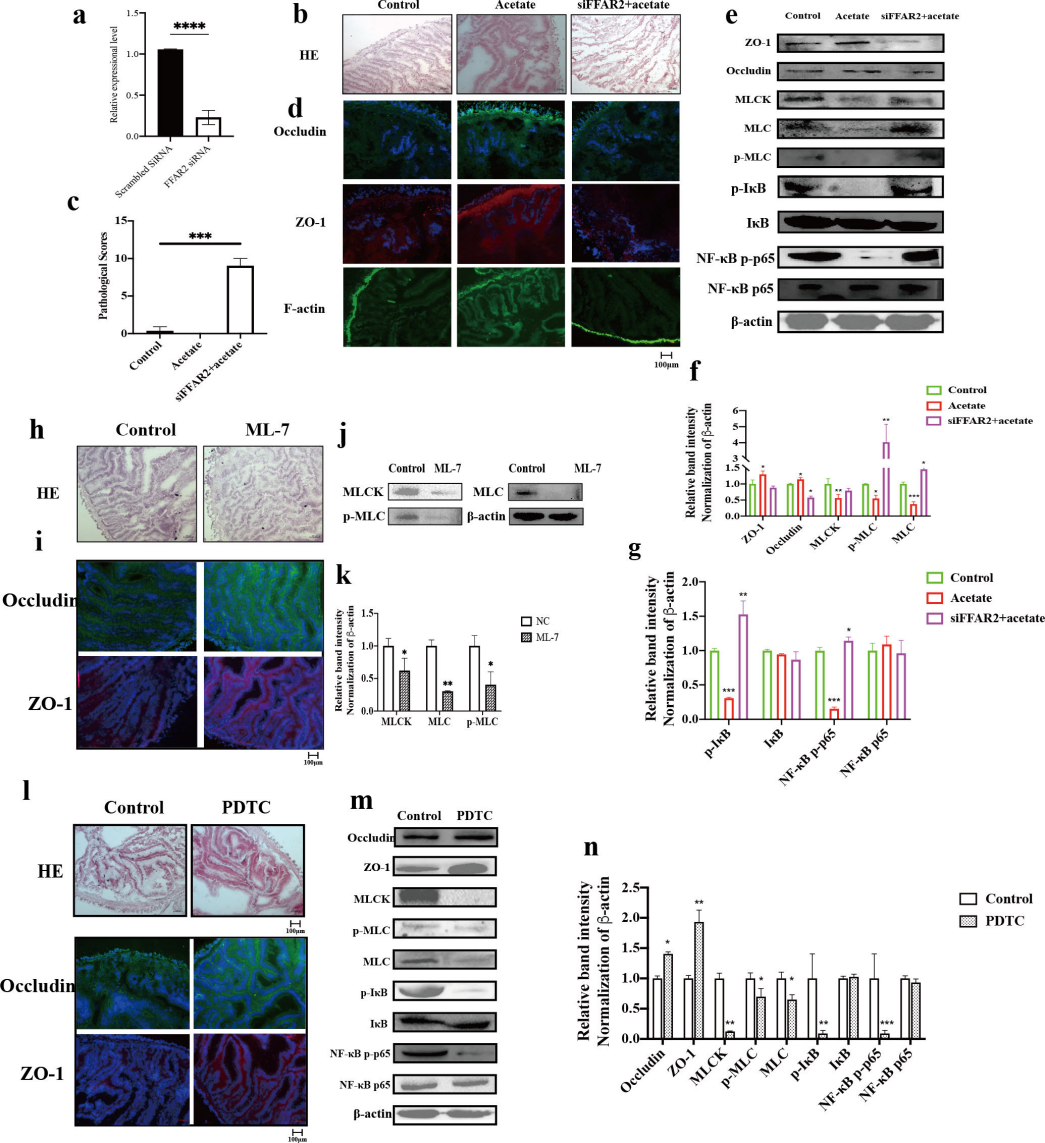
The regulation of acetate on intestinal barrier function via NF-κB–MLCK–MLC signal pathway. (a) The siRNA FFAR2 interference efficiency was detected using qRT-PCR. (b) The effect of FFAR2 knockdown on intestinal morphology. (c) Pathological scores evaluate the siRNA FFAR2 damage on intestines. (d) The immunofluorescence of Occludin and ZO-1. (e) Western blotting of Occludin, ZO-1, and NF-κB–MLCK–MLC signaling molecules. (f) Quantification of western blotting results. (g) Quantification of western blotting results. (h) The effect of ML-7 on intestinal morphology. (i) The immunofluorescence of Occludin and ZO-1. (j) ML-7 repressed the activation of MLCK–MLC signal pathway. (k) Quantification of MLCK–MLC signaling western blotting results. (k) Quantification of NF-κB signaling molecular expression. (l) The effect of PDTC on intestinal morphology and immunofluorescence of Occludin and ZO-1. (m) PDTC repressed the expression of tight junction proteins and NF-κB–MLCK–MLC signaling moleculars. (n) Quantification of NF-κB–MLCK–MLC signaling molecular expression (*, *P* < 0.05; **, *P* < 0.01; ***, *P* < 0.001).

**Fig 6:**
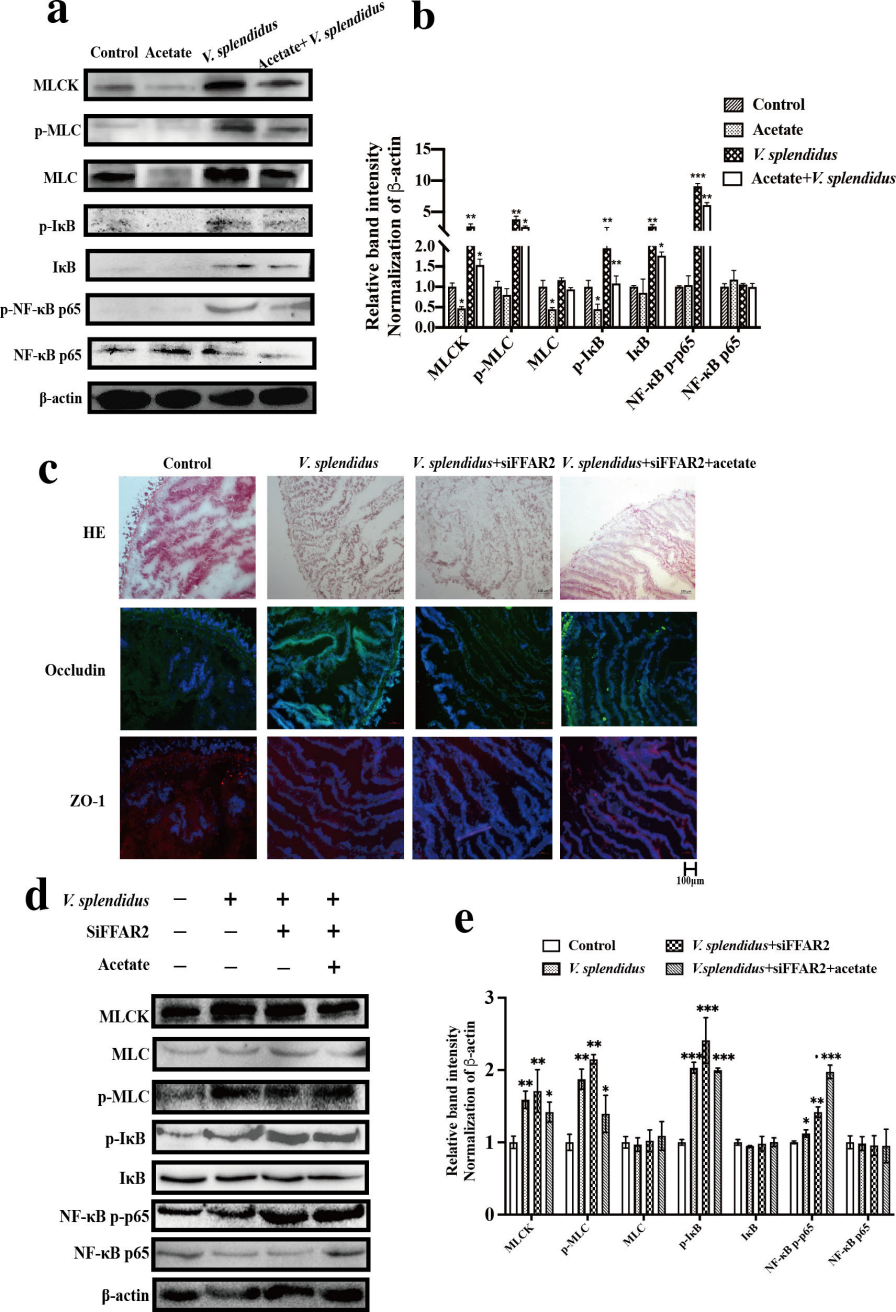
NF-κB–MLCK–MLC signal pathway affects tight junction arrangement. (a) The western blotting results of NF-κB–MLCK–MLC signaling molecules show acetate inhibition NF-κB–MLCK–MLC signal pathway activated *V. splendidus*. (b) Quantification of western blotting results. (c) Intestinal morphology and immunofluorescence of Occludin and ZO-1 results show acetate exerts protective effect on intestine barrier function in a FFAR2-dependent manner. (d) NF-κB–MLCK–MLC signaling molecular expression in *A. japonicus* intestine treated with siRNA FFAR2, *V. splendidus*, and acetate. (e) Quantification of western blotting results (*, *P* < 0.05; **, *P* < 0.01; ***, *P* < 0.001).

Our previous work has demonstrated that acetate could reorganize TJ structure and increase the expression level of TJ proteins to improve intestinal barrier function (24). However, the regulating mechanism of acetate on intestinal barrier is unknown. Given that acetate can specifically recognize and bind to FFAR2, we evaluated the impact of FFAR2 knockdown on intestine (35–38). After FFAR2-specific siRNA transfection for 24 h, the expression of FFAR2 was reduced by 75% (Fig. 5a). The intestine of siFFAR2 *A. japonicus* showed a significant damage in epithelial integrity despite the presence of acetate, compared with healthy and acetate feeding *A. japonicus*, including obvious shedding of the intestine villi and striated borders (Fig. 5b). Pathological score of intestines in siFFAR2 + acetate *A. japonicus* was also significantly higher than that in the control and acetate groups *A. japonicus* (*P* < 0.001) (Fig. 5c). Furthermore, siRNA FFAR2 significantly reduced the expressional level of Occludin and ZO-1 (Fig. 5d through f). In addition, Occludin, ZO-1, and F-actin significantly aggregate toward the nucleus (Fig. 5d). The results of Western blot analysis revealed that siFFAR2 increased the expression level of MLCK, MLC, and p-MLC compared with the control and acetate groups *A. japonicus* (*P* < 0.05) (Fig. 5e and f). TJ structure is known to be regulated by the MLCK–MLC signaling pathway. To confirm the role of MLCK–MLC signaling in TJ structure, *A. japonicus* received ML-7 treatment as the inhibitor of MLCK signaling (Fig. 5h, j and k) ML-7 significantly decreased the expression level and phosphorylation level of MLC (Fig. 5j and k). Intestine villus in the intestine of *A. japonicus* treated with ML-7 was denser than that in control *A. japonicus*, the array of villus was more orderly, and muscle layer was thicker. Occludin and ZO-1 arranged in a linear pattern around the cells in the mucosal layer and were densely distributed in ML-7 treatment (Fig. 5h). Furthermore, the NF-κB signaling molecules were significantly increased induced by siRNA-FFAR2, although NF-κB p-p65 and p-I κB were decreased by acetate (Fig. 5e and g). The results of PDTC treatment, as the inhibitor of NF-κB, suggested that intestinal morphology was similar to that in ML-7 treatment (Fig. 6l). Occludin and ZO-1 were significantly increased by PDTC treatment, while the expression level of MLCK and MLC significantly decreased (*P* < 0.05) (Fig. 6m and n).

The level of NF-κB–MLCK–MLC signaling in *A. japonicus* infected by *V. splendidus* was evaluated to further testify whether acetate could exert a protective effect on intestinal barrier via the FFAR2–NF-κB–MLCK–MLC axis. As shown in Fig. 6a and b, *V. splendidus* significantly increased the expression level of MLCK, MLC, and p-MLC (*P* < 0.05), while acetate significantly decreased them (*P* < 0.05). And the expression level of MLCK, MLC, and p-MLC could be reduced to a lower level which is consistent with that in control under acetate treatment. Meanwhile, *V. splendidus* significantly increased the expression level of IκB and p-IκB (*P* < 0.05), and acetate could repress NF-κB signaling molecules. Next, we analyzed the impact of *V. splendidus* on intestine transfected by siFFAR2. The Fig. 5d control images for Occludin and ZO-1 are being reused in Fig. 6c for clarity and ease of comparison. As shown in Fig. 6c, siFFAR2 transfection significantly exacerbated the damage of *V. splendidus* on the intestine although in the presence of acetate. The TJ proteins of intestine also tended to aggregate towards the nucleus in *V. splendidus*, *V. splendidus* + siFFAR2, and *V. splendidus* + siFFAR2 + acetate (Fig. 6c). SiFFAR2 transfection distinctly increased the expression of MLCK–MLC and NF- κB signaling molecules compared with the control group (*P* < 0.05) (Fig. 6d and e). The abovementioned results suggested that the protection of acetate occurred by acetate binding to FFAR2, given that acetate had no beneficial effect in siFFAR2 *A. japonicus*.

## DISCUSSION

**Fig 7:**
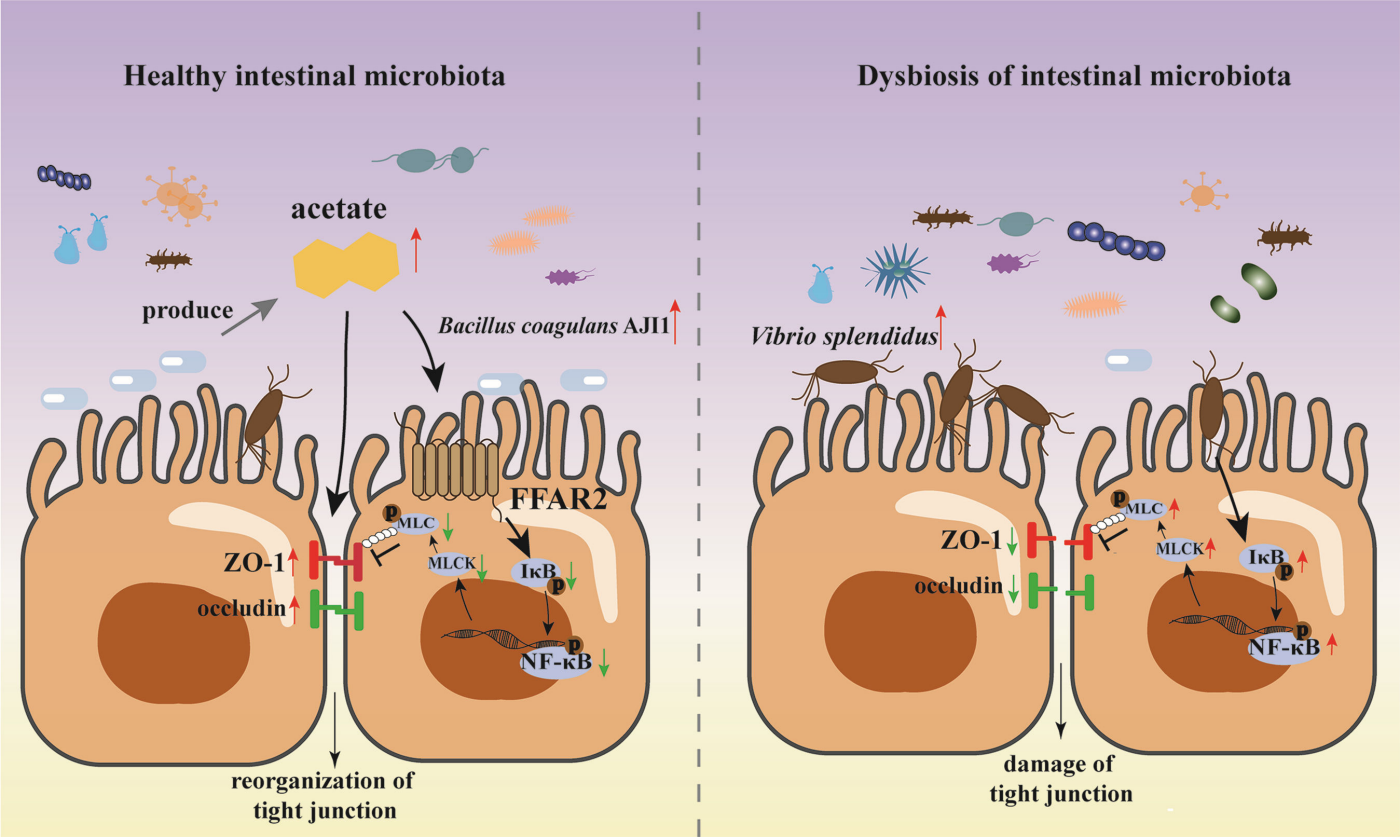
The schematic diagram shows the mechanisms underlying acetate regulating tight junction via suppression of FFAR2–NF-κB–MLCK–MLC axis. Healthy *A. japonicus* intestine possesses an IM composition structure homeostasis. *B. coagulans*, which is an acetate-producing bacteria, maintains intestinal barrier function via inhibitory regulation of acetate on FFAR2–NF-κB–MLCK–MLC axis. *V. splendidus* invasion disrupts the homeostasis of gut microbial community structure and causes abundance of SCFAs-producing bacteria to decrease acetate in content. The acetate/FFAR2–NF-κB–MLCK–MLC pathway is significantly upregulated, leading to signaling is significantly upregulated, leading to the destruction of tight junction structures and a decrease in tight junction protein expression. Thus, the intestinal barrier function is severely damaged, thereby promoting disease occurrence (red arrows indicate upregulation of expression, green arrows indicate downregulation of expression).

The IM is known to regulate the host health in higher vertebrates (39, 40). However, the research involved in the IM of invertebrates mainly focused on IM dysbiosis and probiotics application. In the study, we first established the IMT model in *A. japonicus* to explore the interaction between IM and host health and the causal link between IM dysbiosis and disease development. Our results suggested that IMT can successfully remodel IM community structure and further reveal the regulatory and protective role for the IM in *A. japonicus* intestinal barrier function via SCFAs during the SUS pathogenesis. Importantly, colonization of GF and *V. splendidus* infected *A. japonicus* with an acetate-producing bacterium, which was isolated from healthy *A. japonicus* intestine shows apparent effects on strengthening the intestinal barrier function. Furthermore, we found that acetate mediates intestinal barrier integrity by inhibiting the FFAR2–NF-κB–MLCK–MLC signaling, which can be activated by *V. splendidus* (Fig. 7). This study is the first to validate the relationship of dysbiotic IM in sea cucumber disease, which exemplifies the regulation of IM on disease development. In particular, acetate, as a link between IM and host, has been shown to modulate intestinal health in *A. japonicus*. Our studies support that microbiota have been a potent regulator of SUS disease and intestinal homeostasis in *A. japonicus*.

The intestinal microbiome is a complex and dynamic ecosystem that tends to a state of equilibrium. However, such a microbial balance can be disrupted in its structure and functional capabilities by invading pathogens or pathobionts (41, 42). Our previous studies have indicated that IM dysbiosis occurred in SUS-diseased *A. japonicus* (23, 24). The dysbiotic IM in recipients can be retrieved from transplantation from SUS-diseased *A. japonicus*, which leads to similar symptoms and histological pathology as in SUS-diseased *A. japonicus* (Fig. 1). Several studies have reported that the altered microbial composition is inconsistent among individuals and stages after fecal microbiota transplantation (12, 43). In this IMT experiment, we compared the anal cavity and oral cavity methods of IM mixture injection and ultimately chose the oral injection method of IM mixture. Notably, the dysbiotic composition remains stable in the newly SUS-diseased *A. japonicus.* The characteristics of IM dysbiosis in the first and subsequent IMT involve a decrease in α-diversity, the loss of beneficial bacteria diversity, and the over-growth of harmful microorganisms, which is consistent with the IM composition in SUS-diseased *A. japonicus* (Fig. 2). Molecular ecological networks and null community modeling were conducted to further investigate the community assembly patterns of the causal role of IM dysbiosis in SUS. Clear stratification networks were observed between healthy and diseased *A. japonicus*, which suggests that *A. japonicus* disease substantially disturbed the balance of interspecies interaction (Fig. 2i). Here, metabolite profile, as another common feature, is similar in all SUS individuals. The increase in environmental niche in the gut and decrease in antimicrobials further promote the invasion and colonization of enteric pathogen IM, which escalates the severity of SUS (44). Considerable evidence suggests that IM dysbiosis could lead to several complex diseases in higher vertebrates (3, 5). However, the resistance of intestinal bacteria against pathogens cannot be ignored. The mechanism of IM for pathogen colonization is antagonism, which is prominently characterized by the production of antimicrobials and metabolites to compete for environmental niche and modify host microenvironmental conditions (10). Therefore, we can harness the resistance of IM community for *V. splendidus* colonization to control SUS. The results of reverse IMT revealed that the dysbiotic IM in recipients can be restored to normal microbial structure by transplantation from healthy *A. japonicus*, which leads to the restoration of intestinal barrier function in *A. japonicus*. However, SUS-diseased *A. japonicus* was not cured by IMT from healthy donors. Thus, our study revealed a close and indivisible relation between SUS and IM. Accumulating evidence suggests that IMT is a proven highly effective treatment for various diseases, which provides a new strategy to cure SUS of *A. japonicus* as well (45).

We focused on the SCFAs derived from microbiota, which exerts a significant effect on the maintenance of intestinal homeostasis, to better understand the mechanism of interaction between microbiota and host (21). In our previous study, we have demonstrated that *V. splendidus* infection could lead to a decrease in SCFAs in *A. japonicus*, and a decrease in production of SCFAs may result in the reduction in mucus thickness/degradation of the mucus barrier (24). The decrease of SCFAs content was also observed in the first and subsequent IMT, which is consistent with the SCFAs pattern, except for valeric acid and isovaleric acid under *V. splendidus* infection. SCFAs can provide energy to the intestinal epithelium and intestinal barrier (6). Furthermore, the promoting effect of SCFAs on the expression of TJ proteins is a dose-dependent manner (46). The recipients of *A. japonicus* transplanted with healthy IM also showed an increase in Occludin and ZO-1 levels. In the study, acetate could alleviate the damage of *V. splendidus* on intestinal barrier function, as a characteristic metabolite in *A. japonicus* gut. Previous studies related to human intestinal diseases have proven that the bacteria-producing butyric acid, *Clostridium*, are important for intestinal homeostasis by producing butyric acid (47). Targeted SCFA-producing bacterial can be colonized in patients for therapy sepsis (14). To further verify the protective effect of acetate on intestinal barrier, we successfully established an intestinal-germ-free model of *A. japonicus*, and isolated a bacterial-producing acetate, *B. coagulans* AJI1. In regard to producing SCFAs, the genus *Bacillus* is considered to be relatively common SCFA-producing bacteria (48). Although the ability of *B. coagulans* to produce SCFAs is rarely reported, *B. coagulans*, as a kind of probiotic, has been widely used in aquaculture production due to its beneficial effects on intestinal health (49). Our results also suggested that the colonization of *B. coagulans* AJI1 not only promotes intestinal acetate synthesis but also significantly restores the expression level of TJ proteins and intestinal epithelium integrity in SUS-diseased and GF *A. japonicus. Bacillus subtilis* has developed into a food-grade probiotic with the ability to produce butyric acid and has also been used as an animal feed additive to improve growth performance and immunity (48). However, our conclusion was only based on our observations with specific *B. coagulans*. Whether it is commensals or other species in *Bacillus* genus act in *A. japonicus* remains to be investigated.

Finally, we intended to explore the molecular mechanism by which acetate mediates intestinal barrier function. SCFAs exert a protective role in the maintenance of a gastrointestinal epithelial barrier, the regulation of hormone secretion, and the inhibition of enteric inflammation via the combination of FFAR2 (50–52). Recently, several studies found that the activation of FFAR2 can directly bind to and block phosphorylation and degradation of IκBα and finally lead to the inhibition of NF-κB activity (53, 54). NF-κB signaling serves as an effector mechanism of SCFAs, which suggests a pathway through which interaction of SCFA interactions affect the host (4, 55). Acetate induced a positive effect on the expression of Occludin and ZO-1 and a negative effect on NF-κB signaling, which can be reversed by FFAR2 siRNA (Fig. 5). Therefore, these results suggested that the interaction between acetate and NF-κB signaling is induced by FFAR2. Acetate could downregulate the expression of MLCK and phosphorylation level of MLC via inhibiting the transfer of NF-κB p65 from cytoplasm to nucleus. Anterior gradient protein 2 homolog (AGR2) significantly improves TJ structure in IBD patients and this protective mechanism is promoted by the suppression of NF-κB p65 mediated activation of the MLCK–MLC signaling pathway (56). Shi et al. (57) also demonstrated that the downregulation of MLCK signaling molecules induced by inhibition of NF-κB p65 can restore TJ of fish gill. *V. splendidus* could initiate MLCK transcription and increase the expression level of MLCK by activating NF-κB p65 to damage intestinal barrier. MLCK alters the contractility of cells’ cytoskeleton and redistribution of TJ proteins to increase intestinal permeability. The cytoskeleton is mainly composed of microfilaments, microtubules, and intermediate filaments, where microfilaments are closely related to the maintenance of TJ structures (58). The cytoskeleton can participate in the barrier function of epithelial cells in intestinal tissues. Under certain pathological factors, the increase in actin contraction and tension can cause the remodeling of the cytoskeleton and the redistribution of TJ proteins. These phenomena lead to the destruction of TJs and an increase in intestinal mucosal permeability.

In summary, we first explained the causative role of dysbiosis IM in the occurrence of SUS in *A. japonicus* via IMT. Our study provides evidence that IM improves intestinal health in *A. japonicus* through SCFAs, especially acetate. Acetate ameliorates histopathological changes in the intestine and recovers SUS or IM dysbiosis-induced TJ structure, which is attributable to the role of FFAR2 signaling plays in blocking NF-κB signaling activation. The suppression of NF-κB signaling inhibits the expression of MLCK–MLC signaling molecules to increase the expression of Occludin and ZO-1 and restore intestinal barrier function. These results from the current study established a method for preventing SUS via restoring the IM community. SCFAs play a role in improving of intestinal health through the FFAR2–NF-κB–MLCK–MLC pathway.

## MATERIALS AND METHODS

### Experimental animal

Adult *A. japonicus* (200 ± 10 g) was collected from the coast of Yantai, Shandong, China. The animals were acclimated in seawater at 16 ± 1°C for 2 weeks prior to treatment and were fed once per day.

### IMT procedure in *A. japonicus* and sampling

After acclimation, IMT experiments followed the methods developed in zebrafish and shrimp (59, 60). Intestinal samples from healthy *A. japonicus* and SUS-diseased *A. japonicus* were selected as the control donors (*n* = 6) and SUS donors (*n* = 6) at a ratio of 1 donor *A. japonicus* intestine/2 recipient *A. japonicus*. Intestine sample was placed into a sterile tube containing 600 mL of sterile PBS and was then homogenized thoroughly. The mixture was centrifuged at 800 rpm for 1 min and the supernatant was transferred into a new centrifuge tube. The supernatant was also filtered through a 0.22-µm filter to collect the IM filtrate without bacteria. *A. japonicus* received IM by IMT following previous reports (60). Using a pipette, 1 mL of mixture was introduced in the *A. japonicus* oral cavity dispensing the IM mixture slowly to avoid traumatic effects. Starting 12 h after the IMT process, the *A. japonicus* was fed two times a day.

Control *A. japonicus* was selected as recipients for four groups (*n* = 10 *A. japonicus* per group): IMT by using (I) PBS (C + P1); (II) IM from SUS donors (C + S1); (III) IM from control donors (C + C); and (IV) IM filtrate without bacteria from SUS donors (C + WS). To reveal whether the dysbiotic IM composition was stable in *A. japonicus* suffering from SUS, six *A. japonicus* in the C + S1 group were chosen to be SUS donors for the subsequent IMT. *A. japonicus* were randomly distributed to two groups (*n* = 10 *A. japonicus* per group): IMT with (I) PBS (C + P2) and (II) IM from C + S1 SUS donors (C + S2). For further evidence of the relationship between IM and SUS, the SUS-diseased *A. japonicus* was received IMT from the health *A. japonicus* donors. The reverse IMT was conducted after the abovementioned IMT experiment. The SUS-diseased *A. japonicus* was assigned to two groups (*n* = 8 *A. japonicus* per group): IMT with (I) PBS (S + P); (II) IM from control donors (S + C). Recipient *A. japonicus* transferred with IM were kept feeding once per day until recipients *A. japonicus* developed SUS symptoms. Intestinal content was collected and stored at −80°C for IM and SCFA profile analysis.

### High throughput sequencing of 16S rRNA gene

Total genomic DNA was extracted using DNA Extraction Kit following the instructions of the manufacturer. Concentration of DNA was verified with NanoDrop and agarose gel. The genome DNA was used as template for PCR amplification with the barcoded primers and Tks Gflex DNA Polymerase (Takara). The primer pair 343F (5′-TACGGRAGGCAGCAG-3′ and 798R (5′-AGGGTATCTAATCCT-3′) were used to amplify the V4 region of 16S rRNA gene. Amplicon quality was visualized using gel electrophoresis, purified with AMPure XP beads (Agencourt), and amplified for another round of PCR. After purification with the AMPure XzP beads again, the final amplicon was quantified using a Qubit dsDNA assay kit. Equal amounts of purified amplicon were pooled for subsequent sequencing. Illumina MiSeq or NovaSeq sequencing was conducted to generate raw data. After downloading the data, first use cutadapt software was used to cut out the primer sequence from the raw data sequence. Then, using DADA2, the qualified dual-ended raw data from the previous step will be subjected to quality filtering, noise reduction, splicing, and chimerism removal analysis according to the default parameters of QIIME 2 (2020.11) to obtain representative sequences and ASV abundance tables. Raw sequencing data were in FASTQ format. Paired-end reads were then preprocessed using cutadapt software to detect and cutoff the adapter. After trimming, low-quality sequences were obtained by filtering paired-end reads, denoising, merging, and detecting and cutting off chimera reads using DADA2 with the default parameters of QIIME2 (2020.11). Finally, at last, the software outputs the representative reads and the ASV abundance table. The representative read of each ASV was selected using QIIME2 package. All representative reads were annotated and blasted against Silva database Version 138 (or Unite) (16S/18S/ITS rDNA) using q2-feature-classifier with the default parameters.

### Intestinal content collection and targeted SCFAs profiling

*A. japonicus* intestinal content of 50 mg was harvested for LC–MS/MS analysis. An appropriate amount of the sample was taken, and 300 µL of 50% acetonitrile aqueous solution (vol/vol, containing internal standards [2H9]—Pentanoic acid and [2H11]—Hexanoic acid) (pre-cooled at 4°C) was added. The sample was ground for 3 min (with the sample tray pre-cooled at −20°C) and extracted using ultrasound in an ice water bath for 10 min. Centrifugation was performed for 10 min at 4°C and 12,000 rpm). The supernatant was diluted five times with 50% acetonitrile aqueous solution (vol/vol, containing internal standard [2H9]—Pentanoic acid and [2H11]—Hexanoic acid). An 80 µL aliquot of the supernatant was transferred to an injection vial, followed by the addition of 40 µL of 200 mM 3-NPH (50% acetonitrile aqueous solution configuration, vol/vol) and 40 µL of 120 mM EDC-6% pyridine (50% acetonitrile aqueous solution configuration, vol/vol). The reaction was conducted at 4°C for 30 min. After cooling on ice for 1 min, 160 µL of the supernatant was aspirated and filtered with a 0.22-µm organic phase pinhole filter. The filtered solution was transferred to a brown injection bottle and stored at −80°C until analysis on the machine. An 80 µL aliquot was taken in the injection glass bottle of L standard. Then, 40 µL of 200 mM 3-NPH (50% acetonitrile aqueous solution configuration, vol/vol) and 40 µL of 120 mM EDC-6% pyridine (50% acetonitrile aqueous solution configuration, vol/vol) were added. The reaction was conducted at 40°C for 30 min. After cooling on ice for 1 min, 160 µL of supernatant was aspirated using a syringe. The solution was then filtered with a 0.22-µm organic phase pinhole filter and transferred to a brown injection bottle, which was stored at −80°C. The metabolic profiles were analyzed on a Nexera UHPLC LC-30A (Shimadzu, JP). The injection volume was 1 µL, with a constant flow rate of 0.4 mL/min through the column. The mobile phase consisted of A: 0.1% formic acid aqueous solution and B: acetonitrile/methanol = 2:1. The gradient elution procedures were as follows: 0 min A/B (80:20, vol/vol), 2 min A/B (80:20, vol/vol), 8 min A/B (60:40, vol/vol), 8.1 min A/B (5:95, vol/vol), 9.5 min A/B (5:95, vol/vol), 9.6 min A/B (80:20, vol/vol), and 10 min A/B (80:20, vol/vol). The curtain air pressure was 35 psi; collision-activated dissociation was used; negative ion spray voltage was set to −4,500 V; ion source temperature was 450°C; column temperature was 40°C; and gas1 and gas2 pressures were both 50 psi.

### Isolation of single bacterial strains from *A. japonicus* IM

The adult *A. japonicus* was dissected in a sterile environment, and the intestinal tract was placed in sterile physiological saline and ground to prepare an IM suspension. The suspension and a 10 mL seawater sample were added into a 100 mL lactobacilli broth medium (Hopebio, China), which was cultured at 37°C and 160 r/min until reaching medium turbidity. The abovementioned bacterial fluid was gardenly diluted by physiological saline and then coated on MRS agar medium. After 24–72 h of incubation at 28.5°C in anaerobic conditions, colonies on the plates were isolated and purified by streaking three times on MRS plates. Gram staining, microscopic examination, and H_2_O_2_ test were performed on the purified colonies, and Gram-positive strains were selected and stored at −80°C in 50% glycerol for future use.

### Identification of isolated single bacterial strains

Each isolated bacterial strain was regarded as template to amplify the full-length of 16S rRNA gene by PCR with primers 27F (AGAGTTTGATCCTGGCTCAG) and 1492R (AAGGAGGTGATCCAGCCGCA). Amplification was conducted in a 25 µL reaction volume containing 2.5 µL of 10× buffer, 1 µL dNTP, 1 µL each of forward and reverse primers, 0.2 µL of rTaq enzyme, 1 µL bacterial strain, and 17.3 µL of RNase-free water to a total volume of 25 µL. The reaction mixtures were incubated at 95°C for 5 min, followed by 35 cycles of denaturation at 95°C for 5 s, annealing at 55°C and extension at 72°C for 1 min. After amplification reaction, PCR products were added to 1.5% agarose gel spot sampling hole for electrophoresis for 25 min. The successfully amplified PCR products were sent to YouKang Biotechnology Co., Ltd. for sequencing. After proofreading the sequencing results, the 16S rRNA sequences of the isolated bacteria were subjected to blast similarity analysis on NCBI. The 16S rRNA sequences of the strains with higher homology were selected, and a phylogenetic tree was constructed using the adjacency method using MEGA 7.0 software.

### Construction of GF *A. japonicus* model

The *A. japonicus* was soaked for 3 h in sterile filtered seawater containing 100 U/mL penicillin and 100 µg/mL streptomycin. Then, they were cleaned two times in 0.003% sodium hypochloride (61). Thereafter, *A. japonicus* was transferred to sterile filtered seawater tanks at a density of 3 *A. japonicus* per tank in a sterile room as the GF group; the tanks remained sterile for the duration of the experiment.

### Colonization GF *A. japonicus* with mono-associations

GF *A. japonicus* was colonized with a single bacterial species, and the isolated bacterial strains were cultured, respectively, overnight in YPD 37°C. The suspension of each strain was then centrifuged at 7,000 rpm for 10 min to remove the supernatant, followed by three times washing with sterile PBS buffer. The pellet was then resuspended with sterile filtered seawater and applied to the GF *A. japonicus* at a final concentration of 10^5^ CFU/mL.

### Histopathological analysis and immunofluorescence

For histopathological analysis, an abdominal incision of *A. japonicus* was made to expose its internal cavity and facilitates the separation of the midgut. Then, the midgut was cutoff using small surgical scissors at each sampling time point as described above. Midgut tissues from different treated *A. japonicus* were fixed with 4% paraformaldehyde and embedded in paraffin. Sections (5 µm thickness) were stained with hematoxylin and eosin (H&E). The images were acquired using a light microscope (Carl Zeiss, German). For midgut tissue immunofluorescence analysis, *A. japonicus* midgut was placed in an optimal cutting temperature compound within 5 min. Especially, 10 µm sections were cut, fixed in 4% paraformaldehyde for 15 min, washed three times in 1× PBS, permeabilized in 1× PBS containing 0.5% Triton X-100 for 20 min, washed three times in 1× PBS, blocked for 30 min in antibody diluent, and stained with primary antibodies ZO-1 and Occludin (1:200, Beyotime Biotechnology, China) for 30 min in antibody diluent. After three washes, sections were stained with secondary antibodies Alexa Fluor 555 labeled goat-anti-rabbit lgG (1:1,000, Biotechnology, China) and Alexa Fluor 488 labeled goat-anti-rabbit lgG (1:1,000, Biotechnology, China) for 30 min. After three washes, the nucleus was stained using DAPI (1:10,000, Beyotime Biotechnology, China) for 5 min. Sections were mounted and images were acquired using a Zeiss confocal microscope (Carl Zeiss, LSM880, Germany).

### FFAR2 knockdown

SiRNA targeting FFAR2 (sense: 5′-GUCCUCAUCUUGUCCAAUATT-3′, anti-sense: 5′-UAUUGGACAAGAUGAGGACTT-3′) or control siRNA (sense: 5′-UUC UCCGAACGUGUCACGUTT-3′, anti-sense: 5′-ACGUGACACGUUCGGAGAA TT-3′) was synthesized by GenePharma (Shanghai, China). The transfection operation was performed in *A. japonicus*. According to the instructions for Lipo6000 Transfection Reagent (Beyotime, Shanghai, China), 80 µL PBS, 10 µL transfection reagent, and 10 µL siRNA-FFAR2 were added into 1.5 mL sterile centrifuge tube, mixed and left at room temperature for 5 min, and then injected in an *A. japonicus* of the siRNA-FFAR2 group. The mixture, including 80 µL PBS, 10 µL transfection reagent, and 10 µL stable negative control siRNA, was injected in control *A. japonicus* as control group. The control group also called as scrambled siRNA group in qPCR experiment for detecting interference efficiency. Besides, the control group was used both for subsequent acetate and for *V. splendidus* treatment experiment on siRNA-FFAR2 *A. japonicus*. Then, the intestine of *A. japonicus* was collected and determined by qPCR, Western blotting analysis, histopathological analysis, and immunofluorescence.

### Western blot

Midgut tissues of 100 mg were washed two times with cold PBS and lysed in a 1 mL cold lysis buffer. After incubated at −20°C overnight, the lysates were centrifuged at 1,000 rpm for 20 min at 4°C. The level of total proteins was determined using the BCA assay kits according to the protocol of the manufacturer. After boiling the samples for 10 min, equal amounts of protein (50 µg) were separated by sodium dodecyl sulfate–polyacrylamide gel electrophoresis (SDS-PAGE) and electrophoretically transferred to nitrocellulose filter (NC) membranes. Blocking was conducted by incubating the membranes for 2 h at room temperature with 5% nonfat, dry milk diluted in TBST containing 0.1% Tween-20. The NC membranes were then incubated overnight with primary antibodies against ZO-1 (1:2,000, Beyotime Biotechnology, China), Occludin (1:2,000, Beyotime Biotechnology, China), or β-actin (1:5,000, Proteintech Group, USA) at 4°C with gentle shaking. After washing three times with TBST, the membranes were incubated with HRP-conjugated goat-anti-rabbit IgG (Beyotime Biotechnology, China) diluted 1:5,000 for 1 h at room temperature. After the last washing in a triple with TBST for 5 min, the immunoreactive proteins were investigated with the Omega Lum C imaging system (Aplegen, California, USA). Bands were quantified with the ImageJ software, and the gray value was indicated as statistical analysis of three independent experiments.

### Statistical analysis

Statistical analyses were performed using SPSS V23.0 statistical software (SPSS Inc., Chicago, IL, USA). All data were expressed as mean ± standard deviation. Comparison between groups was performed using two-sided unpaired *t* test if there were more than two groups were involved, and differences among the three groups were tested using one-way analysis of variance with Duncan’s range tests. Correlations were analyzed using Pearson’s correlation. The differences were considered as significant at *P*  <  0.05.

## Data Availability

The 16S rRNA gene sequencing data used in this study are available in the NCBI Short Read Archive (https://www.ncbi.nlm.nih.gov/sra) under Bioproject PRJNA1047199.

## SUPPLEMENTAL MATERIAL

Supplemental material msystems.00602-24-s0001.docx Figures S1 and S2 and Table S1.

ASM does not own the copyrights to Supplemental Material that may be linked to, or accessed through, an article. The authors have granted ASM a non-exclusive, world-wide license to publish the Supplemental Material files. Please contact the corresponding author directly for reuse.
