# Supplementary material for: Bacillus coagulans restores pathogen-induced intestinal dysfunction via acetate–FFAR2–NF-κB–MLCK–MLC axis in Apostichopus japonicus
Source: mSystems. 2024 Jun 28;9(7):e00602-24. doi: 10.1128/msystems.00602-24 (PMC11265352; doi:10.1128/msystems.00602-24)
Supplement: Supplemental material — Figures S1 and S2 and Table S1. [file msystems.00602-24-s0001.docx]

**Supplementary materials**

Figure S1. Identification of *Bacillus coagulans* AJI1 from *A. japonicus* intestinal microbiota. a. Strain *B. coagulans* AJI1 was identified Gram-positive. b. The catalase test of *B. coagulans* AJI1is positive. c. The V-P test of *B. coagulans* AJI1 is positive. d. The methyl red t of *B. coagulans* AJI1 is positive. e. The gelatin liquefaction test of *B. coagulans* AJI1is positive. f. The phylogenetic tree of *B. coagulans* AJI1 based on 16S rRNA gene, the tree was constructed using the Neighbor-Joining method in MEGA7.


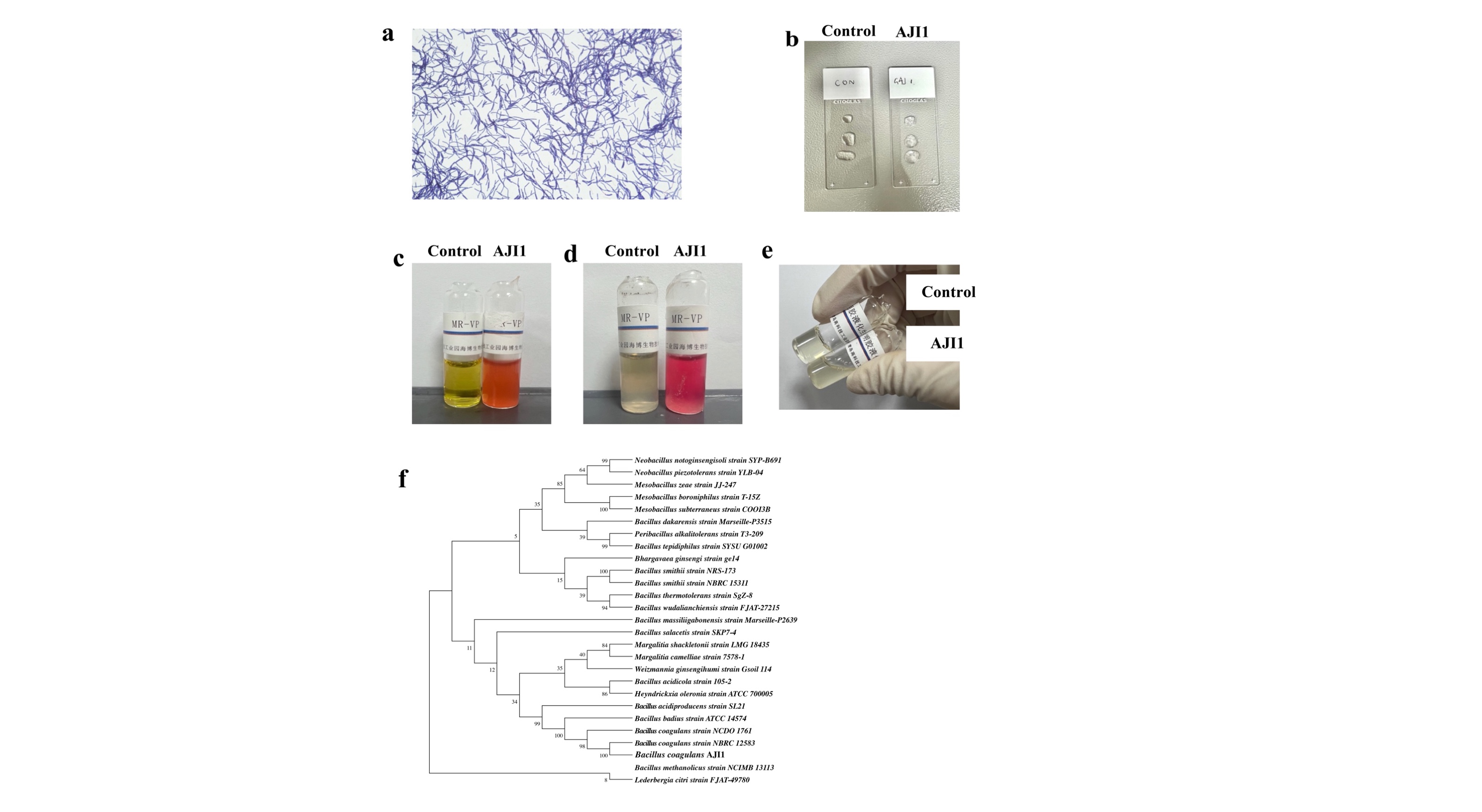


Figure S2. Construction of germ free (GF) *A. japanicous* model and colonization GF *A. japonicus* with *B. coagulans* AJI1 treatment. a. Sterility test of intestine in GF *A. japanicous* by culturing intestine in Luria broth (LB) b. Sterility test of intestine in GF *A. japanicous* by culturing intestine in LB agar plates. c. Quantification of 16S rRNA gene copies in GF and control *A. japanicous.* d. Quantification of the colony forming units (CFUs) per intestine for *B. coagulans* AJI1 to control and GF *A. japanicou,* mean ± SEM of three independent experiments. e.


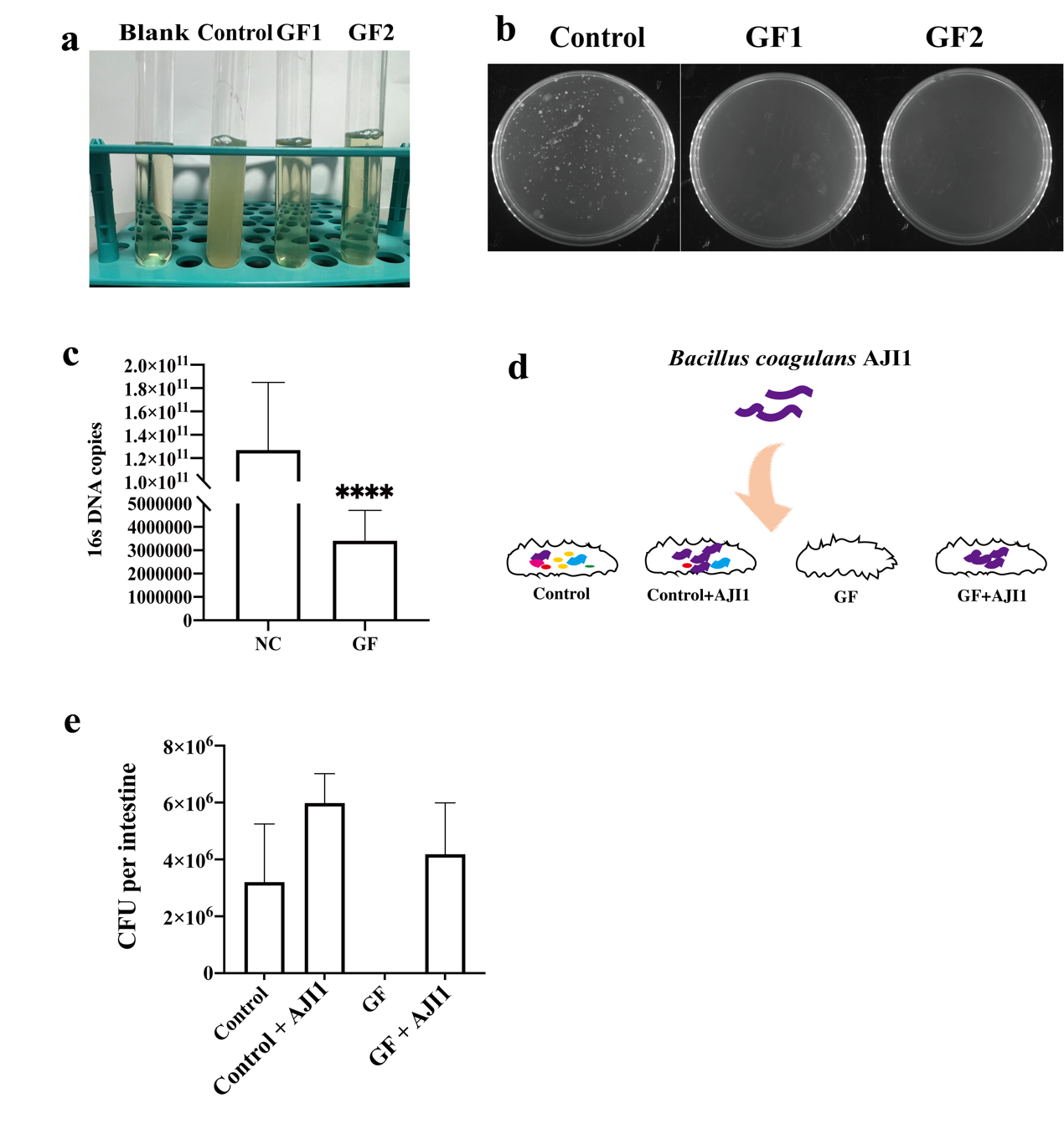


| IMT | Group | 0h | 12h | 24h | 36h | 48h | 60h | 72h | 84h | 96h | 108h | 120h | Total |
| --- | --- | --- | --- | --- | --- | --- | --- | --- | --- | --- | --- | --- | --- |
| first IMT | C+P1 | 0 | 0 | 0 | 0 | 0 | 0 | 0 | 0 | 0 | 0 | 0 | 0 |
|  | C+S1 | 0 | 0 | 0 | 1 | 2 | 2 | 2 | 0 | 0 | 0 | 0 | 7 |
| subsequent IMT | C+P2 | 0 | 0 | 0 | 0 | 0 | 0 | 0 | 0 | 0 | 0 | 0 | 0 |
|  | C+S2 | 0 | 0 | 1 | 0 | 2 | 0 | 0 | 2 | 0 | 0 | 0 | 5 |

Table S1 The numbers of SUS-developed *A. japonicus* at different time points after IMT.
